# Uncertainty quantification via localized gradients for deep learning-based medical image assessments

**DOI:** 10.1088/1361-6560/ad611d

**Published:** 2024-07-19

**Authors:** Brayden Schott, Dmitry Pinchuk, Victor Santoro-Fernandes, Žan Klaneček, Luciano Rivetti, Alison Deatsch, Scott Perlman, Yixuan Li, Robert Jeraj

**Affiliations:** 1 Department of Medical Physics, School of Medicine and Public Health, University of Wisconsin, Madison, WI, United States of America; 2 Faculty of Mathematics and Physics, University of Ljubljana, Ljubljana, Slovenia; 3 Department of Radiology, Section of Nuclear Medicine, School of Medicine and Public Health, University of Wisconsin, Madison, WI, United States of America; 4 Department of Computer Sciences, School of Computer, Data, & Information Sciences, University of Wisconsin, Madison, WI, United States of America

**Keywords:** deep learning, uncertainty quantification, tumor segmentation, model trustworthiness, model reliability, gradient information, metastatic disease

## Abstract

**Objective.:**

Deep learning models that aid in medical image assessment tasks must be both accurate and reliable to be deployed within clinical settings. While deep learning models have been shown to be highly accurate across a variety of tasks, measures that indicate the reliability of these models are less established. Increasingly, uncertainty quantification (UQ) methods are being introduced to inform users on the reliability of model outputs. However, most existing methods cannot be augmented to previously validated models because they are not post hoc, and they change a model’s output. In this work, we overcome these limitations by introducing a novel post hoc UQ method, termed *Local Gradients UQ*, and demonstrate its utility for deep learning-based metastatic disease delineation.

**Approach.:**

This method leverages a trained model’s localized gradient space to assess sensitivities to trained model parameters. We compared the Local Gradients UQ method to non-gradient measures defined using model probability outputs. The performance of each uncertainty measure was assessed in four clinically relevant experiments: (1) response to artificially degraded image quality, (2) comparison between matched high- and low-quality clinical images, (3) false positive (FP) filtering, and (4) correspondence with physician-rated disease likelihood.

**Main results.:**

(1) Response to artificially degraded image quality was enhanced by the Local Gradients UQ method, where the median percent difference between matching lesions in non-degraded and most degraded images was consistently higher for the Local Gradients uncertainty measure than the non-gradient uncertainty measures (e.g. 62.35% vs. 2.16% for additive Gaussian noise). (2) The Local Gradients UQ measure responded better to high- and low-quality clinical images (*p* < 0.05 vs *p* > 0.1 for both non-gradient uncertainty measures). (3) FP filtering performance was enhanced by the Local Gradients UQ method when compared to the non-gradient methods, increasing the area under the receiver operating characteristic curve (ROC AUC) by 20.1% and decreasing the false positive rate by 26%. (4) The Local Gradients UQ method also showed more favorable correspondence with physician-rated likelihood for malignant lesions by increasing ROC AUC for correspondence with physician-rated disease likelihood by 16.2%.

**Significance.:**

In summary, this work introduces and validates a novel gradient-based UQ method for deep learning-based medical image assessments to enhance user trust when using deployed clinical models.

## 1. Introduction

### 1.1. Overview

Deep learning models yield incredible performance across a variety of medical image assessment tasks. Given the remarkable milestones achieved by these models, such as comparable performance to human experts (Liu et al 2019), the large-scale clinical deployment of deep learning models appears imminent. Most deep learning models, however, are missing a critical and necessary component for responsible and reliable clinical use—uncertainty quantification (UQ).

The outputs of deep learning models inherently contain uncertainty, which is often unaccounted for in standard models. This lack of uncertainty information can lead to severe consequences, especially when relying on deep learning models to inform high-stakes clinical decisions. Despite its critical importance, UQ appears to be insufficiently studied. This is likely due in part to the complexity of the task as the probability values of contemporary deep learning models are innately poorly calibrated, meaning they deviate from a reliable representation of task accuracy (Guo et al 2017). As a result, the models may make highly confident yet incorrect decisions. Thus, auxiliary methods are required to quantify deep learning model uncertainty.

One example of a deep learning task in critical need of UQ is metastatic cancer delineation, which allows for the identification of disease extent, its heterogeneity, and treatment response assessment. As over 90% of cancer patients die due to metastatic disease (Chaffer and Weinberg 2011), monitoring the behavior of individual metastatic lesions is critical for effective management of cancer patients (Harmon et al 2017, Kyriakopoulos et al 2020). Several studies have investigated the utility of deep learning models for automatic metastatic malignancy delineation (Li et al 2020, Weisman et al 2020, Liu et al 2021, Weber et al 2021, Bilic et al 2023, Schott et al 2023). These models output a voxel-wise disease mask, which in turn, can be used for a variety of downstream clinical tasks. For instance, in (Weber et al 2021, Schott et al 2023, Lokre et al 2024, Santoro-Fernandes et al 2024) the deep learning-based disease delineations were used to extract image biomarkers, which were in turn used as inputs for a predictive model of patient outcome to treatment. The used delineation models, however, do not provide uncertainty information. Without uncertainty information of the predicted delineations, the accuracy of any downstream clinical assessment derived from the disease masks cannot be ensured.

UQ is critical for metastatic cancer delineation due to the unique image interpretation challenges involved in this task. First, due to a variety of biological factors, disease presentation can vary greatly within and across patients (Sica et al 2000, Perk et al 2018b, Ozaki et al 2022). As a result, even tumors in close proximity to each other may differ in presentation and conspicuity. Second, healthy physiology may mimic diseased regions, depending on the disease type and imaging modality (Reinking and Osman 2009, Kuyumcu et al 2013). Lastly, certain benign pathologies may appear very similar to malignant tumors, which are often the target of the delineation task (Delbeke et al 1998, Even-Sapir et al 2006). All of these image interpretation challenges introduce added complexity and uncertainty to the learning process of a deep learning model. Thus, UQ methods are especially needed for this task.

The uncertainty of deep learning models stems from a variety of sources including input data noise (aleatoric uncertainty), poor training procedures, inadequate model architecture, and insufficient knowledge of the data space (epistemic uncertainty). Several methods exist to estimate these uncertainties. Simple approaches have been investigated such as using the maximum (Hendrycks and Gimpel 2017) or the Kullback–Leibler (KL) divergence (Lee et al 2018, Hendrycks et al 2019) of a model’s *softmax* probability output. More involved methods exist such as the Bayesian-based Monte Carlo (MC) dropout method, where dropout layers are used during model training and inference (Gal and Ghahramani 2016). Deep ensembling methods infer on a given test sample using multiple independently trained models (Lakshminarayanan et al 2017). Similarly, test-time data augmentation techniques infer on a given test sample multiple times through a single trained model, yet with different data augmentations applied each time (Seçkin Ayhan and Berens 2018). For MC dropout, deep ensembles, and test-time data augmentation techniques, an uncertainty measure can be obtained by leveraging the prediction distribution from the multiple forward passes through the trained network(s). Finally, several methods act on the outputs of a deterministic model such as by applying model re-calibration techniques (Guo et al 2017, Kuleshov et al 2018, Tomani et al 2021), by computing the output probability entropy (Rottmann and Schubert 2019), or by leveraging specialized model gradient information (Huang et al 2021), among others.

Several studies have begun implementing UQ methods for medical image analysis tasks. Among these medical image studies, reporting the maximum *softmax* probability as a baseline is common (DeVries and Taylor 2018, Berger et al 2021, González et al 2022). The entropy of predicted probabilities has been used for UQ in structure delineation tasks for PET, CT, and MRI data (Mehrtash et al 2020, Diao et al 2022). (Ghesu et al 2021) developed a novel model derived from the Dempster–Shafer theory of evidence was formulated to output uncertainty values for classification tasks across a variety of image modalities. It was shown that test samples with high uncertainty values degraded classification performance. In (G. Wang et al 2019), test-time data augmentation was used to generate uncertainty measures that corresponded with delineation performance in MRI data. MC dropout-based methods similarly have been used to flag poorly segmented test images (McClure et al 2019, Mehrtash et al 2020, Nair et al 2020, Klanecek et al 2023, Ng et al 2023). Following the logic behind the Deep Ensembles, (Kushibar et al 2022) constructed ensembled delineation outputs from various layers within a single network. The variation of a segmented region across layer outputs was used as an uncertainty measure, which again was shown to correspond with delineation quality. Most closely related to the metastatic disease delineation application, (Nair et al 2020) used MC dropout to generate uncertainty values for detecting and segmenting Multiple Sclerosis lesions imaged on MRI and demonstrated the utility of the uncertainty measure to enhance lesion detection performance.

These previously developed UQ methods largely do not meet at least one of two constraints for streamlined clinical implementation. First, most of these methods are not post hoc, meaning that they cannot be implemented on a previously trained model. Rather, their use necessitates complete model re-training. The only method that can be considered post hoc is test-time data augmentation, as implemented in (Wang et al 2019). Second, all of these methods will induce changes to the predictive outputs of a previously trained model. The performance of deployed models will need to be re-validated because of this change in model outputs. UQ methods designed to be post hoc and to not change a previously trained model’s output would be clinically advantageous because they can retrospectively be augmented to deployed models without the need for model re-training or performance re-validation. In addition, many previous UQ methods, such as MC dropout, output voxel-wise uncertainty measures. The acquisition of region-wise uncertainty is important for many tasks such as metastatic disease delineation where a priority of the image analysis is to determine if suspicious lesions are actually malignant or not. Voxel-wise uncertainty measures may be aggregated into a region-wise metric, however, the appropriate aggregation strategy may not be straight-forward.

In this work, we present and validate a novel UQ method designed for deep learning tasks with spatially representative outputs (e.g. anatomical delineations). Unlike the previously established methods listed above, this method is designed to be post hoc and will not change a trained model’s output (e.g. predicted delineations). Thus, this method is more suited for the direct integration into deployed clinical models. In addition, the method directly acquires a region-wise uncertainty measure, bypassing any voxel-to-region aggregation step. The method targets a model’s localized gradient space to derive a regional uncertainty measure. The model’s backwards computational path is redirected such that model gradients are computed with respect to individual regions, localizing gradient information to specific regions. Therefore, we refer to this novel method as the *Local Gradients UQ* method. This is the first UQ method for deep learning-based medical image assessment which utilizes information from a model’s gradient space. While the Local Gradients UQ method can be applied to many medical imaging tasks, we demonstrate its utility for metastatic disease delineation tasks, as one of the more demanding tasks. The performance of each uncertainty measure is assessed in four clinically relevant experiments: (1) response to artificially degraded image quality, (2) comparison between matched high- and low-quality clinical images, (3) false positive (FP) filtering, and (4) correspondence with physician-rated disease likelihoods.

## 2. Methods

### 2.1. Local gradients UQ method

#### 2.1.1. Mathematical framework

The Local Gradients UQ method was designed for deep learning models with spatially representative outputs (e.g. anatomical delineation), as opposed to models with 1-D vector outputs (e.g. a classification model). We first assumed that there is a previously trained deep learning model with learned parameters, *θ*. We also assumed that there is a mechanism to localize individual regions in the model output. For instance, a predicted label map can be used for an anatomical delineation task. The goal of the Local Gradients UQ method is to quantify the sensitivity (or change) of a given localized region with respect to the learned model parameters. Mathematically, this can be expressed as the partial derivative of the localized region (*R*) with respect to the model’s learned parameters as

(1) $$\frac{\text{Δ}R}{\text{Δ}\theta }=\frac{\delta R}{\delta \theta }.$$

To carry-out this computation, it is first necessary to formulate a scalar score that sufficiently describes each localized region, referred to as the *regional target function* (*T_R_*). For this, we selected the KL divergence between the model’s predicted class *softmax* output within a localized region ($p=\left\{p_{i}\right\}$) and a reference distribution $\left(q=\left\{q_{i}\right\}\right)$, normalized by the number of voxels within a given localized region, *N*

(2) $$T_{R}=\frac{1}{N}D_{\text{KL}}(p\;\|\;q)=\frac{1}{N}\sum_{i}^{N}p_{i}\text{log}\;\left(\frac{p_{i}}{q_{i}}\right).$$

The KL divergence is a measure of similarity between *p* and *q*. We set the reference distribution to be uniform $q=\left[\frac{1}{C},\frac{1}{C},\ldots ,\frac{1}{C}\right]\in {\mathbb{R}}^{N}$ of length *N* (number of voxels in region), where *C* is the number of output classes (e.g. *C* = 2 for the binary delineation task). This uniform distribution simulates a region made up of voxels with ambiguous classifications. Therefore, for localized regions dominated by high *softmax* probabilities (high confidence), *T_R_* will be large and vice versa.

Substituting *T_R_* from equation (2) into *R* from equation (1), formulated the sensitivity of a localized region with respect to the model parameters as

(3) $$\frac{\delta T_{R}}{\delta \theta }=\frac{\delta \frac{1}{N}D_{\text{KL}}(p\;\|\;q)}{\delta \theta }=\frac{\delta \frac{1}{N}\sum_{i}^{N}p_{i}\text{log}\;\left(\frac{p_{i}}{q_{i}}\right)}{\delta \theta }.$$

*T_R_* was individually backpropagated for each localized region which populated the gradient information of *θ*. Practically speaking, these gradients were accessed using the ‘backwards hook’ capabilities of the PyTorch deep learning library. Rather than collecting gradient information from all model parameters, which may be computationally expensive, we selected a subset of model parameters, *φ*, from which to retrieve gradient information. Gradient information from the selected model parameters was aggregated into a single scalar via the *L*_1_-norm. The Local Gradients score (LG) for a single localized region *R* within a test image *x* can be written as

(4) $$\text{LG}{\left(x\right)}_{R}={\left\|\frac{\delta T_{R}}{\delta \varphi }\right\|}_{1}={\left\|\frac{\delta \frac{1}{N}\sum_{i}^{N}p_{i}\text{log}\left(\frac{p_{i}}{q_{i}}\right)}{\delta \varphi }\right\|}_{1}$$

where $\|\cdot {\|}_{1}$ denotes the *L*_1_-norm.

To enhance the interpretation of the Local Gradients score, we normalized it to define the Local Gradients uncertainty measure as

(5) $$U{\left(x\right)}_{\text{LG},R}=\frac{\text{LG}{\left(x\right)}_{R}}{P_{95}\left\{{\text{LG}}_{\text{low}}\right\}}$$

where $P_{95}\left\{{LG}_{low}\right\}$ denotes the 95th percentile of a set of Local Gradients scores calculated from model outputs on a validation dataset with a priori assumed low uncertainty. At this percentile, the majority of outputs with assumed low uncertainty are captured while still accounting for potential, high uncertainty outliers.

**Figure 1.**
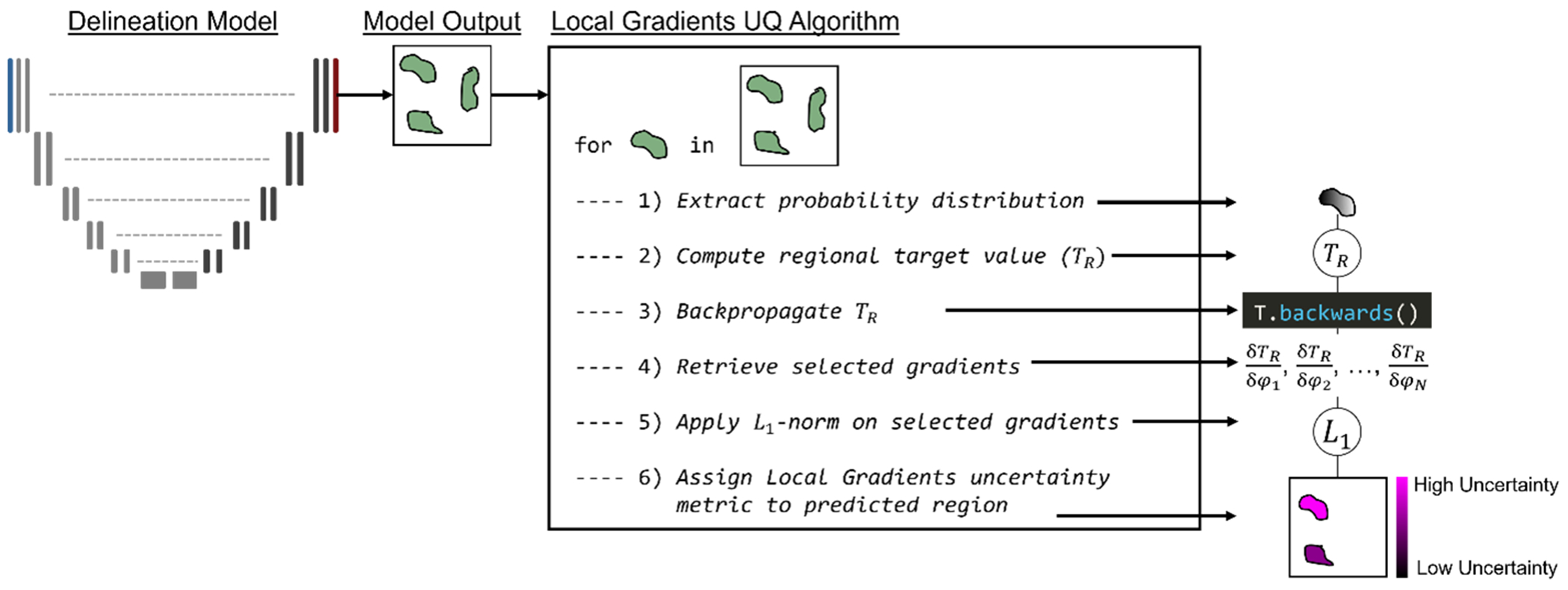
Schematic description of the local gradients UQ algorithm applied to the metastatic disease delineation task. For each delineated (localized) region predicted by the deep learning model, the steps to acquire the uncertainty measure are as follows: (1) extract the softmax probability values within the predicted region, (2) compute the regional target value *T_R_*, (3) backpropagate *T_R_* to populate gradient information on model parameters, (4) retrieve gradient information from the selected model parameters, (5) apply the *L_p_*-norm to aggregate the gradient information into a single value, (6) assign the local gradients UQ measure to the current region to populate the uncertainty map.

Under this formulation, it was assumed that regions with high uncertainty will yield a large gradient response, making *U*(*x*)_LG,*R*_ large, and regions with low uncertainty will yield a small gradient response, making *U*(*x*)_LG,*R*_ small. This process was repeated for each localized region within a given test image. An outline of the Local Gradients UQ algorithm applied to the metastatic disease delineation task is shown in figure 1. A key advantage of the Local Gradients UQ algorithm is that it does not require any image ground-truth data to implement as it only relies on the model’s outputted probabilities (*p*) for a predicted region and a user-defined distribution (*q*) to compare the model probabilities to the *regional target function* (*T_R_*).

#### 2.1.2. Targeted model parameters

**Figure 2.**
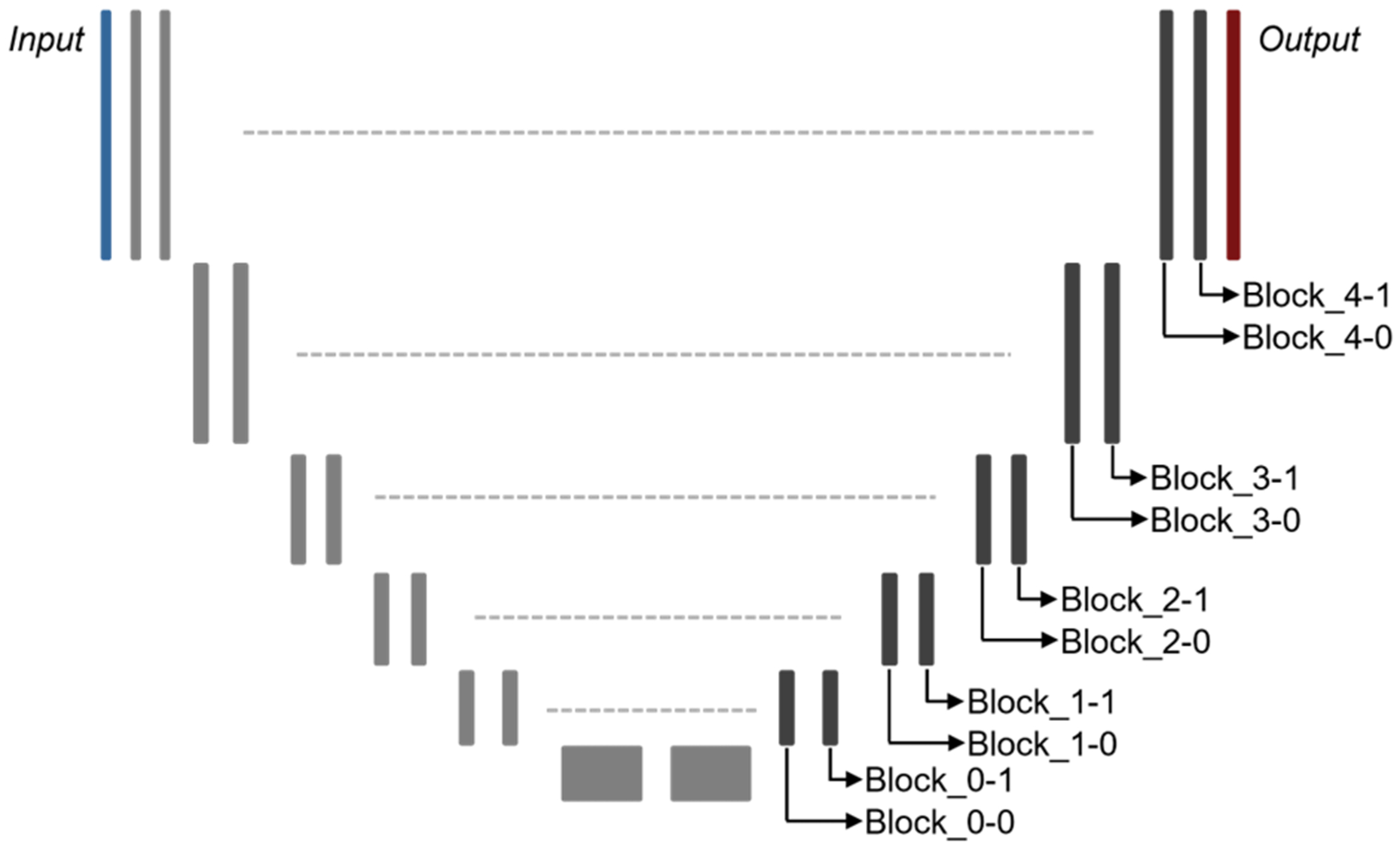
Schematic showing the architecture of the lesion delineation model with decoder convolutional block labeling.

When computing the Local Gradients UQ measure, gradient information can be acquired from any learnable parameter in a trained model. In this work, we used a U-Net model which consists of an encoder, a decoder, and equal-resolution skip connections (Ronneberger et al 2015). Each resolution level of the encoder and decoder contained two convolutional blocks, each consisting of *N* 3D convolutions using a 3 × 3 × 3 kernel, instance normalization, and ReLU activation operations. Both model training and the Local Gradients UQ method were implemented using the PyTorch deep learning library. The Local Gradients UQ measure was obtained by targeting gradient information from all learnable parameters within a given decoder convolutional block. Targeting a U-Net model’s decoder parameters should also capture sensitivities in the mode’s encoder side because encoder activations are passed to the decoder side via skip connections. Thus, we only specifically selected to target decoder parameters in this work. As conveyed in figure 2, we denote the targeted decoder blocks using the resolution level starting from the bottleneck layer (i.e. levels 0–4) and level pair ordering (i.e. pair 0 or 1). For instance, we use ‘Blocks_4-0’ to indicate gradients that are acquired from the first (0, zero-based indexing) convolutional block of the fifth (4) resolution layer of the decoder. In all experiments, parameters from decoder Block_4-0 and Block_4-1 were used for the Local Gradients UQ measure. A sensitivity study was performed in section 3.3 to determine the utility of targeting different decoder block configurations.

#### 2.1.3. Uncertainty measures for comparison

We compared the Local Gradients UQ measure to two measures derived from the *softmax* probability outputs within predicted regions. These measures were selected for comparison because, like the Local Gradients UQ measure, they are post hoc and will not change a trained model’s output and some form of model probability value or entropy have been investigated as UQ baselines previously (Jungo et al 2020, Diao et al 2022). First, we compared to the mean of the *softmax* predicted class probability within a predicted region as in

(6) $$\text{MP}{\left(x\right)}_{R}=\frac{1}{N}\sum_{i}^{N}p_{i}.$$

Second, we compared the Local Gradients UQ measure to using equation 2 on its own, which is simply the KL divergence without any associated gradient information described by

(7) $$\text{KLD}{\left(x\right)}_{R}=\frac{1}{N}\sum_{i}^{N}p_{i}\text{log}\;\left(\frac{p_{i}}{q_{i}}\right).$$

The direction of the uncertainty measure scales in equations (6) and (7) are opposite to the Local Gradients UQ measure, where high magnitudes denote high uncertainties. To account for this difference and to facilitate more interpretable comparisons across all uncertainty measures, we normalized MP(*x*)_*R*_ and KLD(*x*)_*R*_ in the same manner as the Local Gradients UQ measure (equation (5)) and reversed their scales according to

(8) $$U{\left(x\right)}_{\text{MP},R}=-\frac{\text{MP}{\left(x\right)}_{R}}{P_{95}\left\{{\text{MP}}_{\text{low}}\right\}}$$

(9) $$U{\left(x\right)}_{\text{KLD},R}=-\frac{\text{KLD}{\left(x\right)}_{R}}{P_{95}\left\{{\text{KLD}}_{\text{low}}\right\}}.$$

Under this formulation, it is assumed that all uncertainty measures will be small for low uncertainty predictions, and large for high uncertainty predictions. For brevity *U*(*x*)_MP,*R*_, *U*(*X*)_KLD,*R*_, and *U*(*x*)_LG,*R*_ are referred to as, *U*_MP_, *U*_KLD_, and *U*_LG_ in all subsequent experiments, respectively.

### 2.2. Datasets

Two datasets were used to evaluate the performance of the Local Gradients UQ measure. We selected datasets of patients with one of two of the most common malignant metastases sites—liver and bone—which together account for almost half of all metastases (Riihimäki et al 2018). The first dataset consisted of abdominal CT scans of patients with liver metastases and the second dataset consisted of ^18^F-NaF PET/CT scans of patients with bone metastases. The selected datasets are especially relevant for investigating UQ because of their clinical importance. Each dataset also presents unique image interpretation challenges which are likely to induce greater uncertainty. For instance, CT acquisition can be non-standardized between imaging sites, scanners, patient cohorts etc. This non-uniformity is likely to convolute the model fitting process. Similarly, ^18^F-NaF PET/CT scans are often difficult to interpret due to the high incidence of benign uptake in areas of high osteoblastic activity (e.g. arthritic joints) (Even-Sapir et al 2006, Iagaru et al 2012, Sheikhbahaei et al 2019). Thus, the model will be forced to learn the subtle differences between metastatic malignant and benign bone lesions, introducing an added layer of complexity and uncertainty.

#### 2.2.1. Dataset 1—liver metastases

The imaging dataset of liver metastases consisted of both publicly and institutionally available data. The public data was acquired from the LiTS liver tumor segmentation challenge (Bilic et al 2023). This is a diverse dataset consisting of CT scans from seven institutions acquired across a variety of scanners, scan acquisition protocols, and patient metastatic malignant pathologies. All scans were considered diagnostic level with high image resolution and contrast enhancement. We used the 131 available training scans with corresponding ground-truth data. Across all scans, the liver tumors were contoured and independently verified by trained radiologists. In the 131 scans, a total of 908 tumors were contoured (per patient median: 3, range: [0, 75]).

The institutional dataset consisted of patients with metastatic neuroendocrine tumors (NETs) presenting on the liver and was used only for model testing and UQ assessment. All patients were treated at the University of Wisconsin Hospital and Clinics (UWHC) with peptide receptor radionuclide therapy using ^177^Lu-DOTATATE. Prior to and throughout the course of treatment, patients received diagnostic level CT and ^68^Ga-DOTATATE PET/CT imaging for staging and treatment response assessment. We only used the attenuation correction CT scan component of the ^68^Ga-DOTATATE PET/CT scans in our analyses. We refer to the attenuation correction CT scans as the *institutional-attenuation correction* (AC-CT) test set and the diagnostic-level CT scans as the *institutional-diagnostic* (CE-CT) test set. The median time between the acquisition of AC-CT and CE-CT scans for each patient was 4 months (range: [0, 30] months).

#### 2.2.2. Dataset 2—bone metastases

The imaging dataset of bone metastases consisted of institutionally available data only. Patients were acquired from a previously conducted prospective study (NCT01516866) at the UWHC (Lin et al 2016), using ^18^F-NaF PET/CT to assess response in patients with prostate cancer metastasized to bone. For the present work, we retrospectively selected all patient scans acquired before the initiation of a systemic treatment. Scans were acquired across three different sites on either a Discovery VCT (GE Healthcare, Waukesha, WI) PET/CT scanner or a Gemini (Philips Healthcare, Amsterdam, Netherlands) PET/CT scanner. All PET scans were scatter and attenuation corrected, and scans between different scanners were harmonized as in (Jallow 2014, Lin et al 2016).

**Figure 3.**
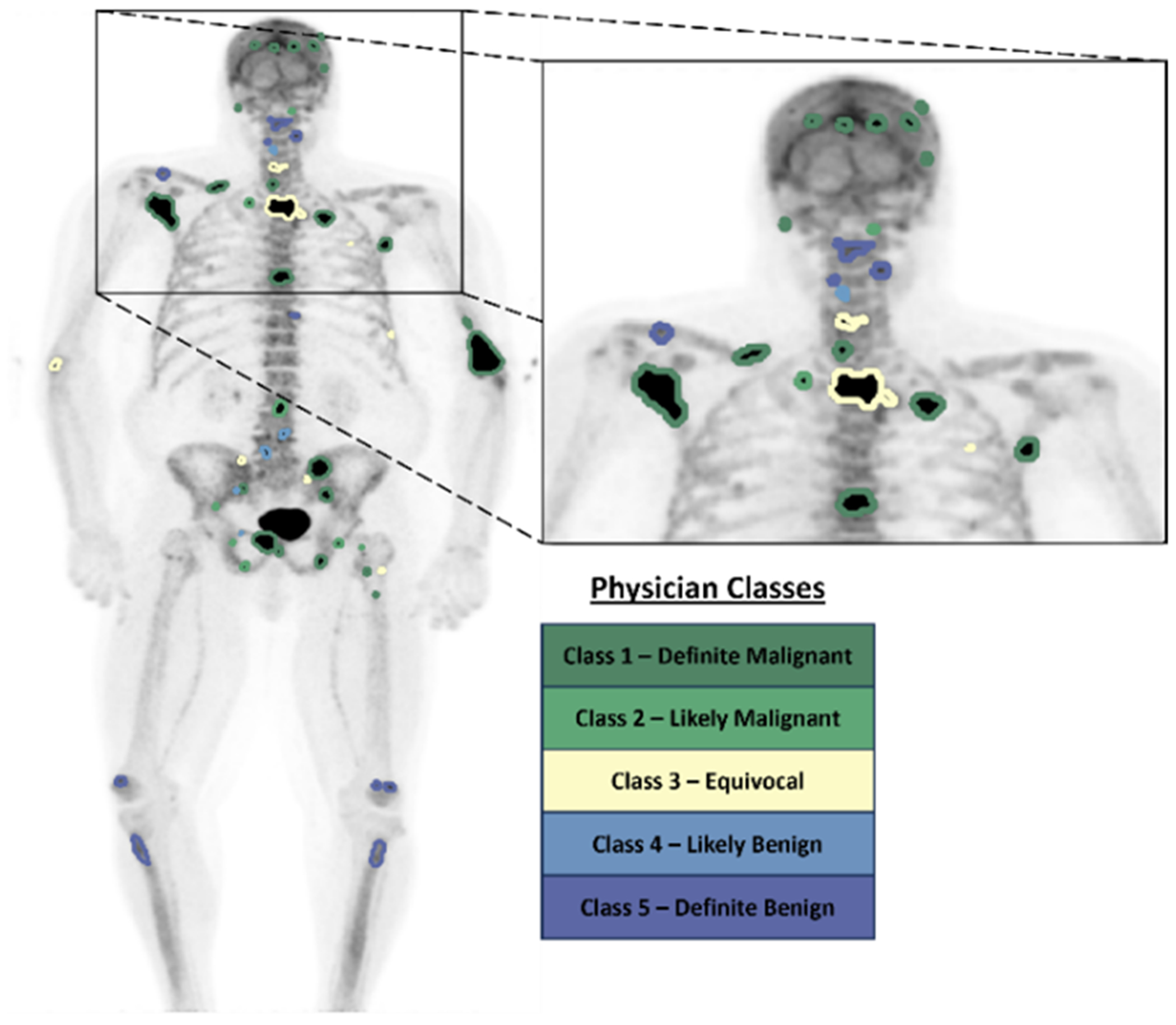
A maximum intensity projection image of an example patient with bone metastases imaged with ^18^F-NaF PET and overlaid physician delineations with disease likelihood classifications.

A total of 37 baseline ^18^F-NaF PET/CT images were acquired of which a nuclear medicine physician manually contoured a total of 1833 bone lesions (per patient median: 42, range: [14, 123]). The same nuclear medicine physician classified all lesions on a five-point scale defined as (1) definitely malignant, (2) likely malignant, (3) equivocal, (4) likely benign, and (5) definitely benign. The distribution of the contoured lesions across the five-classes is summarized in table 1(a). For training the lesion delineation model, these classes were condensed to a 3-point scale, with distributions shown in table 1(b). A maximum intensity projection image of an example ^18^F-NaF PET scan with overlaid physician contours and disease classifications is shown in figure 3.

### 2.3. Lesion delineation model

The Local Gradients UQ method was designed to be implemented on a previously trained model. Thus, we first trained a model for metastatic malignancy delineation in each of the imaging datasets. The two models were trained in-house using the *nnUNet* repository (Isensee et al 2021), a powerful tool for biomedical image delineation which follows a standard U-Net architecture (Ronneberger et al 2015), which is routinely used across a wide variety of medical image delineation tasks. When using *nnUNet*, a subset of the training hyperparameters are automatically and heuristically selected given characteristics of the training data and available computer infrastructure. The *nnUNet* model has achieved highly competitive performance on a variety of delineation tasks (Isensee et al 2021), making it well-suited to assess any UQ method because uncertainty due to poor model design and training will be minimized.

A single, full-resolution 3D model was trained for each dataset and used across all experiments. The model loss function was set as the sum of Dice Coefficient and Cross Entropy, and the model was trained for 1000 epochs using a batch size of 2 and 250 minibatches per epoch. Data augmentation was applied during training across all experiments using the following techniques: rotation, scaling, Gaussian noise, Gaussian blur, brightness, contrast, low resolution simulation, gamma augmentation, and mirroring. The image patch size used during training varied across experiments due to the differences in training data size and resolution. All models were trained on an RTX Titan GPU workstation with 25 GB of memory.

#### 2.3.1. Image pre- and output post-processing

Prior to training, all training images were normalized to zero-mean-unit-variance and were resampled to a common voxel size using linear interpolations. For *Dataset 2—Bone Metastases*, we did not consider lesions that were located in the patients’ hands because the physician did not classify suspicious lesions in the hands. All predicted regions with a volume smaller than 0.25 cm^3^ were removed as in post-processing.

#### 2.3.2. Liver metastases model training

For the experiments on *Dataset 1—Liver Metastases*, we trained an *nnUNet* model to delineate both the liver and liver lesions on CT. 105 of the 131 (80%) of the LiTS images were used for model training. All images were resampled to a common voxel spacing of 1.5 mm^3^, and an image patch size of [112, 128, 160] was used during training. Cross validation was not necessary for this model given the large amount of training data available.

#### 2.3.3. Bone metastases model training

For the experiments on *Dataset 2—Bone Metastases*, we trained an *nnUNet* model for metastatic bone lesion delineation and classification. For this purpose, we collapsed all malignant and benign disease classes (including definite and likely categories) from the five-class physician-rated disease likelihood classifications into a three-class scale defined as: (1) malignant, (2) equivocal, and (3) benign and trained a model to delineate and classify lesions along this three-class scale. The distribution of annotated lesions across these three classes is displayed in table 1(b). Due to the relatively small dataset size, five-fold cross validation was used during training with 80%/20% train/test splits to obtain test predictions on each of the 37 baseline ^18^F-NaF PET/CT scans. All images were resampled to a common voxel spacing of 2.5 mm^3^, and an image patch size of [128, 64, 288] voxels was used during training. The lesion delineation performance of the *nnUNet* model in this dataset has previously been reported in (Schott et al 2023).

### 2.4. Experiments

#### 2.4.1. Response to artificially degraded data

**Figure 4.**
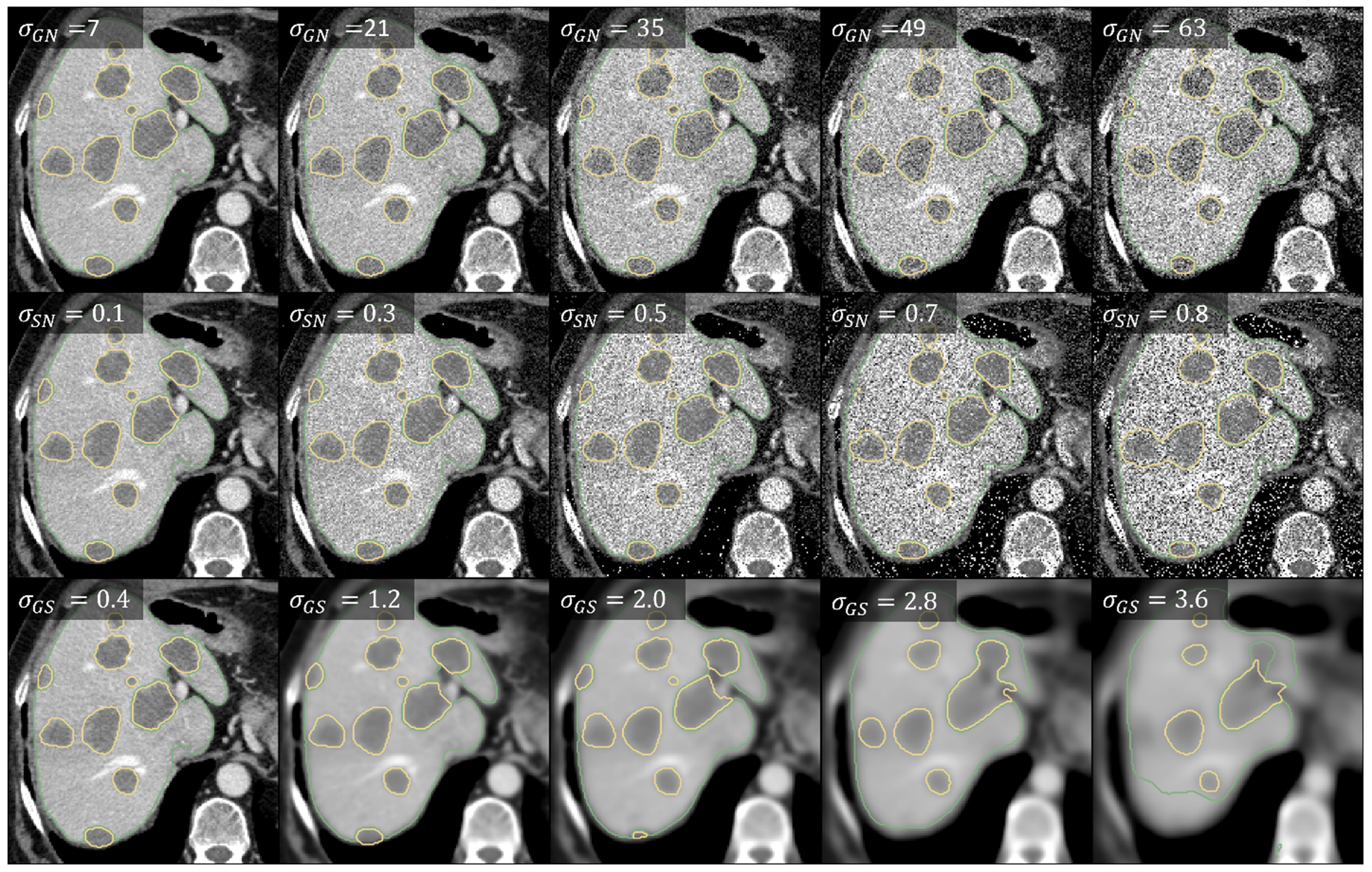
An example test image axial slice across degradation types. Top row: additive Gaussian noise (*σ*_GN_). Middle row: additive speckle noise (*σ*_SN_). Bottom row: Gaussian smoothing (*σ*_GS_). Green and yellow contours indicate predicted liver organ and liver lesion delineations, respectively.

In this first experiment, we investigated the Local Gradients UQ measure’s response to artificially degraded image quality using data from *Dataset 1—Liver Metastases*. Fundamentally, this was implemented as a proof-of-concept study to observe if the uncertainty measures followed expected behavior. We assumed that a reliable uncertainty measure should increase with a decrease in image quality, making the images harder to visually interpret, and consequently the predictions more uncertain. The trained liver lesion CT model was used for inference on the test data split from the LiTS dataset (*N* = 27). For each test image *I ∈* ℝ*^n×m×z^*, artificially degraded images were generated using the following degradation methods with varying magnitudes (*σ*):

1. Additive Gaussian noise: $I_{Noised}=I+W$
2. Additive speckle noise: $I_{Noised}=I+W*I$
3. Gaussian smoothing: $I_{Smoothed}=I⊗K$

where W∼𝒩(μ=0,σ) is an *n × m × z* Gaussian noise matrix with 0-mean and *σ*-standard deviation, K∼𝒩(μ=0,σ) is a Gaussian smoothing kernel with 0-mean and *σ*-standard deviation, *∗* is the point-wise multiplication operation, and *⊗* is the convolution operation. Degradation magnitudes ranged from *σ*_GN_ = [0, 70] voxels for additive Gaussian noise, *σ*_SN_ = [0, 1] voxels for additive speckle noise, and *σ*_GS_ = [0, 4] voxels for Gaussian smoothing, each in 11 steps. Uncertainty information was then computed for each predicted region in all the degraded test images. An example of one test image per degradation type and magnitude is shown in figure 4.

Predicted regions were tracked across the increasing degradation magnitudes for each degradation type using a previously developed lesion tracking tool (Santoro Fernandes et al 2024). This tool constructs matching correspondence between lesions and establishes lesion tracks across the non-degraded and degraded images across all degradation magnitudes for a single test image while accommodating the splitting and merging of lesions. Predicted region uncertainties were acquired for each region in each region track. In the case of a single region splitting into multiple regions at a given magnitude, a weighted average, weighted by region volumes, of the split regions’ uncertainty measures was used. Thus, tracks of uncertainty measures across degradation magnitudes were constructed.

To assess the response of each uncertainty measure as a function of degradation magnitude, the percent difference of uncertainty measures of matched predicted regions across non-degraded and degraded images were computed. This was performed for each degradation type and magnitude on a given test image *x* as

(10) $$\text{Percent}\;\text{Dif}f_{x,R,\sigma =i}=\frac{{\tilde{x}}_{R,\sigma =i}-{\tilde{x}}_{R,\sigma =0}}{{\tilde{x}}_{R,\sigma =0}}\times 100$$

where ${\tilde{x}}_{\sigma =0}$ denotes the undegraded test image, and ${\tilde{x}}_{\sigma =i}$ denotes the degraded test image at degradation magnitude *i*, and *R* denotes the predicted region where they uncertainty is being measured. This relative metric facilitates comparisons of uncertainties at differing degradation magnitude scales. When inferring on test images with increasing degradation magnitude, the delineation model may begin to miss lesions that it previously delineated on less degraded data. Thus, we restricted this analysis to persisting lesions (i.e. lesions that were delineated by the model across all degradation magnitudes).

#### 2.4.2. Comparison on low- and high-quality clinical data

In this second experiment, we again investigated the Local Gradients UQ measure’s response to poor image quality and utilize data from *Dataset 1—Liver Metastases*. Instead of artificially degrading image quality as in the first experiment, we observed the uncertainty measure’s response to lower quality clinical image acquisitions. Again, we assumed that a reliable uncertainty measure should increase with a decrease in image quality. For this experiment, we compared uncertainty measures between matched lesions in the *institutional-diagnostic* (CE-CT) and the *institutional-attenuation correction* (AC-CT) datasets. First, inference was performed on both test datasets and uncertainty measures were computed for each predicted lesion. Subsequently, liver tumors were manually matched across CE-CT and AC-CT scans for each patient and their uncertainties were compared. Anatomical variations in matching tumors across scan types are expected to be minimal since NETs progress very slowly (Dromain et al 2019). Differences in the uncertainty measure between matched CE-CT and AC-CT scans were assessed using statistical significance (Wilcoxon signed rank test).

#### 2.4.3. False positive filtering

In this third experiment, we utilized *Dataset 2—Bone Metastases to* investigate the Local Gradients UQ measure’s ability to filter false positive (FP) from true positive (TP) predicted regions. Following the assumption in (Nair et al 2020), we assumed that FP predicted regions are induced by high predictive uncertainty inherent to the deep learning model. Thus, it is expected that FP regions will have higher uncertainty than TP regions. In our evaluation, a FP region was defined as a predicted region which does not overlap with any corresponding lesion in the ground-truth data. The difference in uncertainty measure magnitude between FP and TP regions was investigated. Statistical significance between uncertainty measures from FP and TP predicted regions was established using the Wilcoxon rank-sum test. Additionally, the area under the receiver operator curve (AUC) and the false positive rate at the 95% sensitivity threshold (FPR95) for distinguishing FP from TP regions using the uncertainty measure were computed.

Within the FP filtering experiment, we performed a sensitivity study to determine the optimal gradient information to leverage when using the Local Gradients UQ method. We investigated FP filtering performance of the Local Gradients UQ method when targeting different configurations of convolutional blocks in the model’s decoder. We performed this sensitivity study using the KL divergence and the mean probability of a predicted region as regional target functions to determine if the selection of the regional target function is critical for the methodology. Using gradient information from the KL divergence calculation, as in figure 1, is equivalent to the Local Gradients UQ measure (*U*_LG_). To use gradient information from the mean probability value within a predicted region, we set *T_R_* = MP(*x*)*_R_* in equation (2) and followed the subsequent steps of the Local Gradients UQ algorithm figure 1. We refer to this modification of the Local Gradients UQ measure as *U*_MP, Gradients_. Finally, both regional target functions were assessed without gradient information (KLD(*x*)*_R_* for KL divergence, as in equation (7), and MP(*x*)*_R_* for mean probability, as in equation (6) to assess the added utility of the gradient space for each regional target function.

#### 2.4.4. Correspondence with physician-rated disease likelihood

In the fourth experiment, we investigated the Local Gradients UQ measure’s correspondence with physician-rated disease likelihood. We utilized the data from *Dataset 2—Bone Metastases*, and we expected the uncertainty measures to follow that of a human observer. For instance, when a human observer is very certain a given region belongs to a given class, the corresponding uncertainty measures of that region should be low. This model used in this experiment was trained to delineate and classify lesions on the three-point scale: (1) malignant disease, (2) equivocal disease, and (3) benign disease. Predicted regions were then grouped according to the corresponding ground-truth classification along the five-point scale of physician-rated likelihood of disease classification (table 1). In the case of multiple classes being matched to a single predicted region, the class with the majority of voxels within a region was selected. Under this setup, it is expected that predicted lesions should yield uncertainty measures in increasing magnitude across physician-rated *definitely, likely*, and *equivocal* disease classes (for both malignant and benign disease groups). Quantitative analysis is performed separately for malignant and benign groups, where the equivocal class is included in both. Differences between each physician-rated class were established using the Wilcoxon rank-sum test, AUC scores, and median percent differences defined as

(11) $${\text{Percent Diff}}_{A,B}=\frac{U_{A}-U_{B}}{U_{B}}\times 100$$

where *U_A_* and *U_B_* denote the uncertainty measures for two arbitrary groups for comparison *A* and *B* (e.g. definitely malignant lesions (*A*) and likely malignant lesions (*B*)).

### 2.5. Uncertainty measure normalization

Each uncertainty measure was normalized according to equations (5), (8) and (9). The normalization constant for each uncertainty measure ($P_{95}\left\{{LG}_{low}\right\},P_{95}\left\{{KLD}_{low}\right\}$, and $P_{95}\left\{{MP}_{low}\right\}$) was acquired from the distribution of uncertainty measures for predicted regions with expected low uncertainty. Normalization constants were specific to each model trained. For the liver metastases model, the normalization constant was defined using the uncertainty measures of the predicted lesions in the non-degraded LiTS validation data. For the bone metastases model, we used the uncertainty measures of the TP predicted regions to define the normalization constant.

## 3. Results

### 3.1. Response to artificially degraded data

The liver metastases model predicted 94 lesions on the 27 non-degraded LiTS validation images. The lesion tracking analysis generated 62, 54 and 29 persistent lesion tracks across all degradation magnitudes for additive Gaussian noise, additive speckle noise, and Gaussian smoothing degradations, respectively.

**Figure 5.**
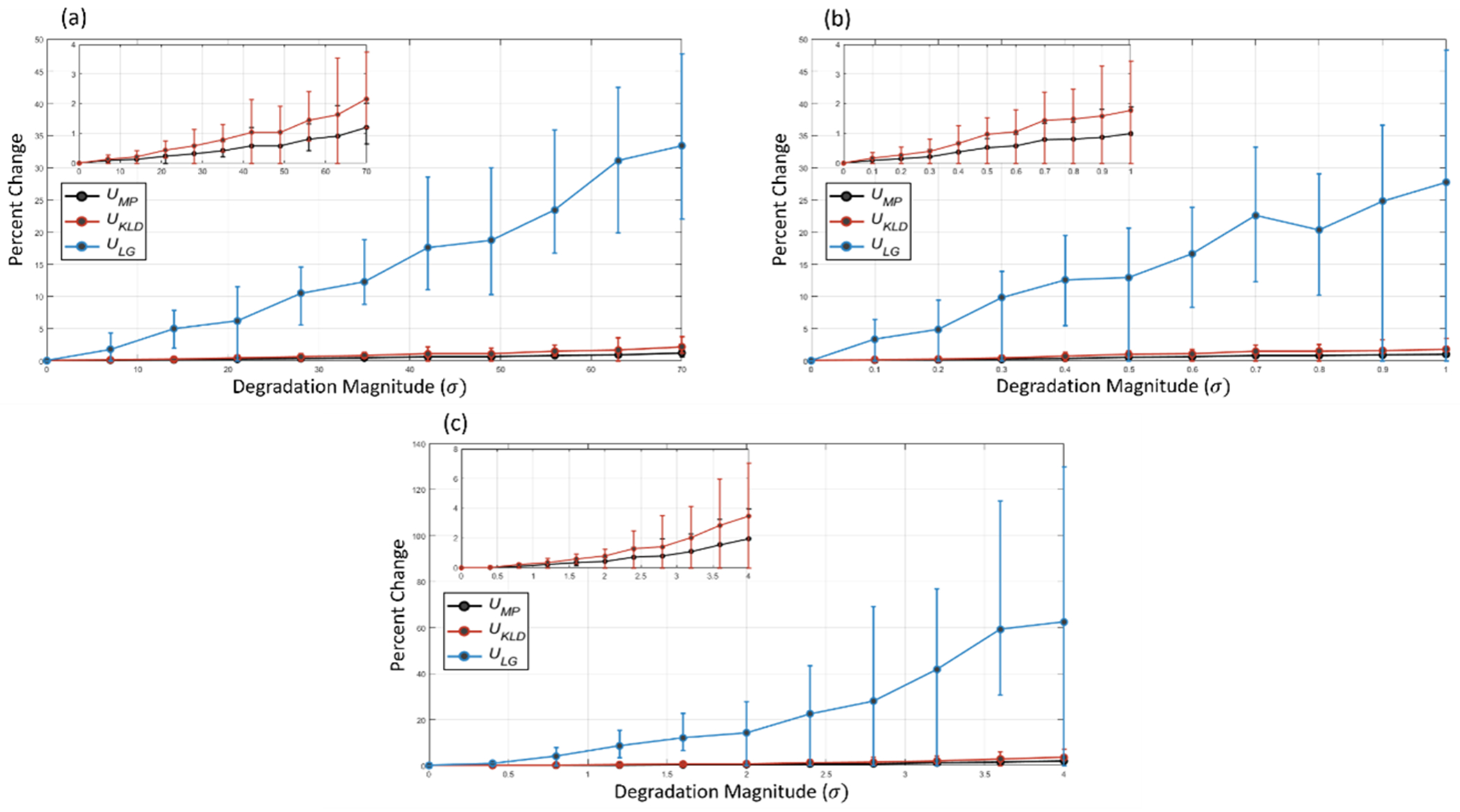
The median percent difference between the uncertainties of predicted regions on non-degraded and degraded images as a function of image degradation magnitude for (a) additive Gaussian noise, (b) additive speckle noise, and (c) Gaussian smoothing degradations. Inset figures show the uncertainty response of the U_MP_ and U_KLD_ measures on a smaller scale. Error bars indicate the interquartile range at each degradation magnitude.

Figure 5 shows the median percent difference between uncertainty measures of matching lesions on non-degraded and degraded images at increasing degradation magnitudes. For all three degradation types, the *U*_KLD_ measure responded stronger to the degraded image data than the *U*_MP_ measure, where the *U*_KLD_ measure median percent difference between the non-degraded and most degraded images was 2.16%, 1.78%, and 3.48% for the additive Gaussian noise, additive speckle noise, and Gaussian smoothing degradations, respectively. The *U*_LG_measure’s response to artificial image degradation was much stronger than both the *U*_MP_ and *U*_KLD_ measures. The median percent differences of the *U*_LG_ measure between the non-degraded images and most degraded images were 33.41%, 27.73%, and 62.35% for additive Gaussian noise, additive speckle noise, and Gaussian smoothing, respectively.

### 3.2. Comparison on low- and high-quality data

In the institutional liver metastases test set, a total of 158 and 78 lesions were delineated by the base CT liver organ and lesion model in the *institutional-diagnostic* (CE-CT) and *institutional-attenuation correction* (AC-CT) test scans, respectively. Of these lesions, 43 pairs were manually matched between CE- and AC-CT image pairs. The Local Gradients UQ measure was calculated for each lesion pair.

**Figure 6.**
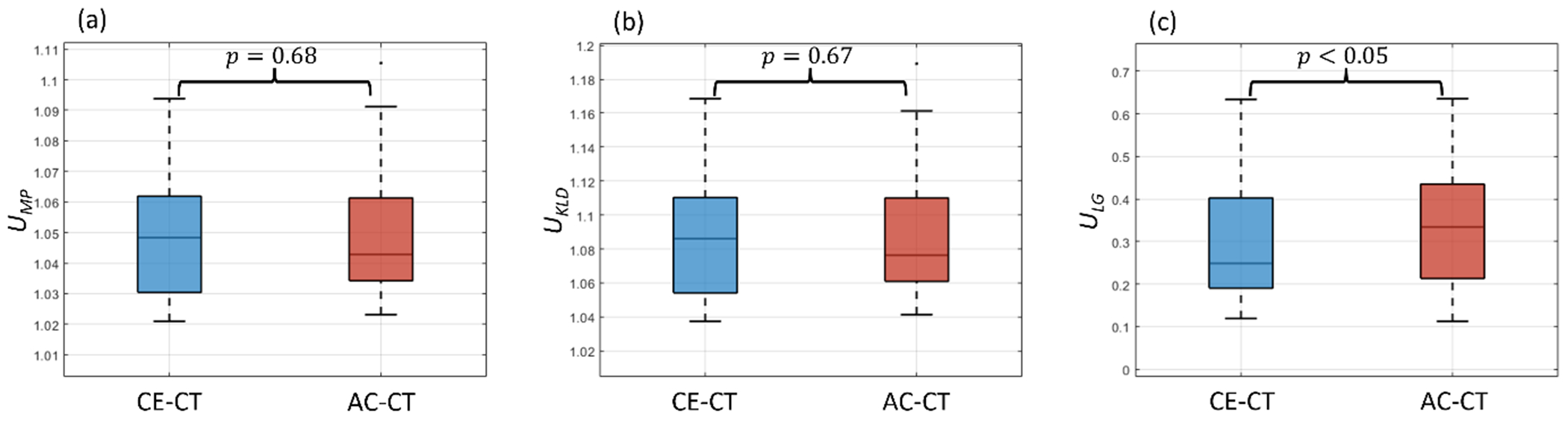
Uncertainty measures of matched predicted liver tumor delineations in high-quality (contrast enhanced CT) and low-quality (attenuation correction CT) medical images. Comparisons between CE-CT and AC-CT are drawn between three uncertainty measures: (a) mean probability (U_MP_), (b) KL divergence (U_KLD_), and (c) local gradients UQ (U_LG_).

The distributions of paired lesions on CE-CT and AC-CT for each uncertainty measures are shown in figure 6. No statistical evidence was found for a difference between predicted lesion uncertainty measures on CE-CT and AC-CT images for the *U*_MP_ and *U*_KLD_ measures. Conversely, the *U*_LG_ uncertainty measure exhibited statistical significance between CE-CT and AC-CT groups (*p* < 0.05), where the AC-CT lesions generally yielded larger uncertainty magnitudes than CE-CT lesions.

**Figure 7.**
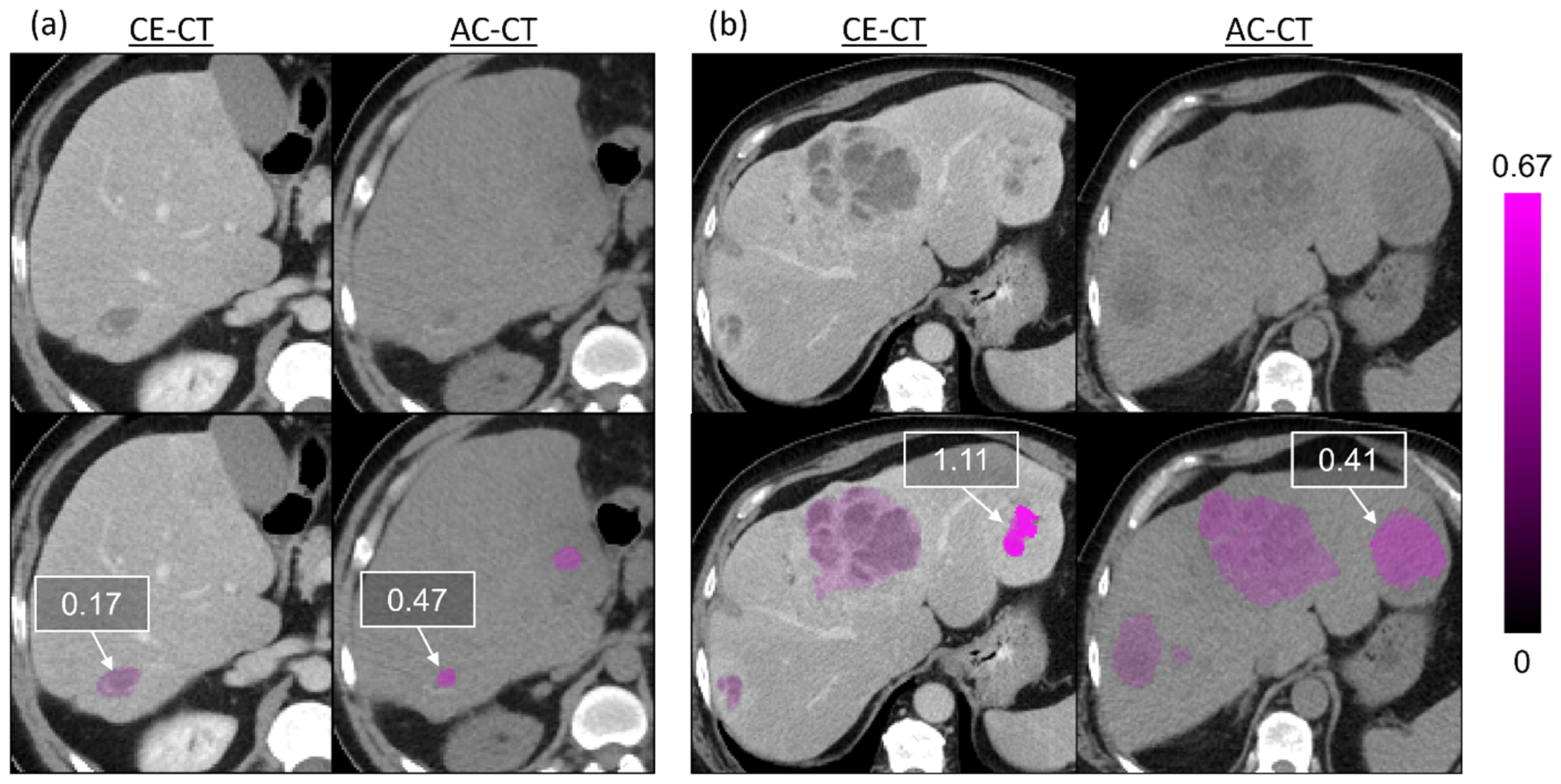
A qualitative evaluation of two example test scans with local gradients UQ measures (U_LG_) overlaid on predicted liver tumor regions. Figure (a) shows an example where the liver tumor is more visible in the CE-CT and has a lower uncertainty measure than the corresponding tumor and uncertainty measure on the AC-CT. Figure (b) shows an example where the uncertainty measure of a predicted tumor on the CE-CT has a higher uncertainty measure than the same tumor on the AC-CT. This discrepancy could be explained by the more heterogeneous presentation of the tumor on the CE-CT than on the AC-CT. U_LG_ uncertainty measure is listed in the box for each lesion. The color bar on the right indicates increasing U_LG_.

In figure 7 we show two scenarios of matched CE-CT and AC-CT lesions with their corresponding *U*_LG_ measures overlaid. Figure 7(a) shows an example with the expected pattern, where the lesions are much more visible on CE-CT than on AC-CT, and the *U*_LG_ measures were higher on CE-CT than on AC-CT. In contrast, figure 7(b) shows an example test scan where the *U*_LG_ measure was higher on CE-CT than on AC-CT for a matched lesion. However, the lesion on CE-CT appears more differentiated than on AC-CT, where it appears larger and more uniform.

### 3.3. False positive filtering

The FP filtering performance of the two tested regional target functions (*T_R_*) with and without gradient information is summarized in table 2. Performance is shown for the different decoder blocks from which gradient information is acquired. We report the performance of each block individually and the combination of each block pair at each level of the decoder (e.g. Block 4–0, Block 4–1).

FP filtering was enhanced with the inclusion of gradient information for both regional target functions (MP(*x*)*_R_* and KLD(*x*)*_R_*) across all convolutional block configurations. When including gradient information, both *U*_MP, Gradients_ and *U*_LG_ achieved comparable FP filtering performance in terms of AUC, where the best AUC was 0.87 for multiple block configurations. *U*_LG_, however, achieved the best FPR95 performance of 0.61 when using gradient information from decoder Block_4-0 and Block_4-1.

**Figure 8.**
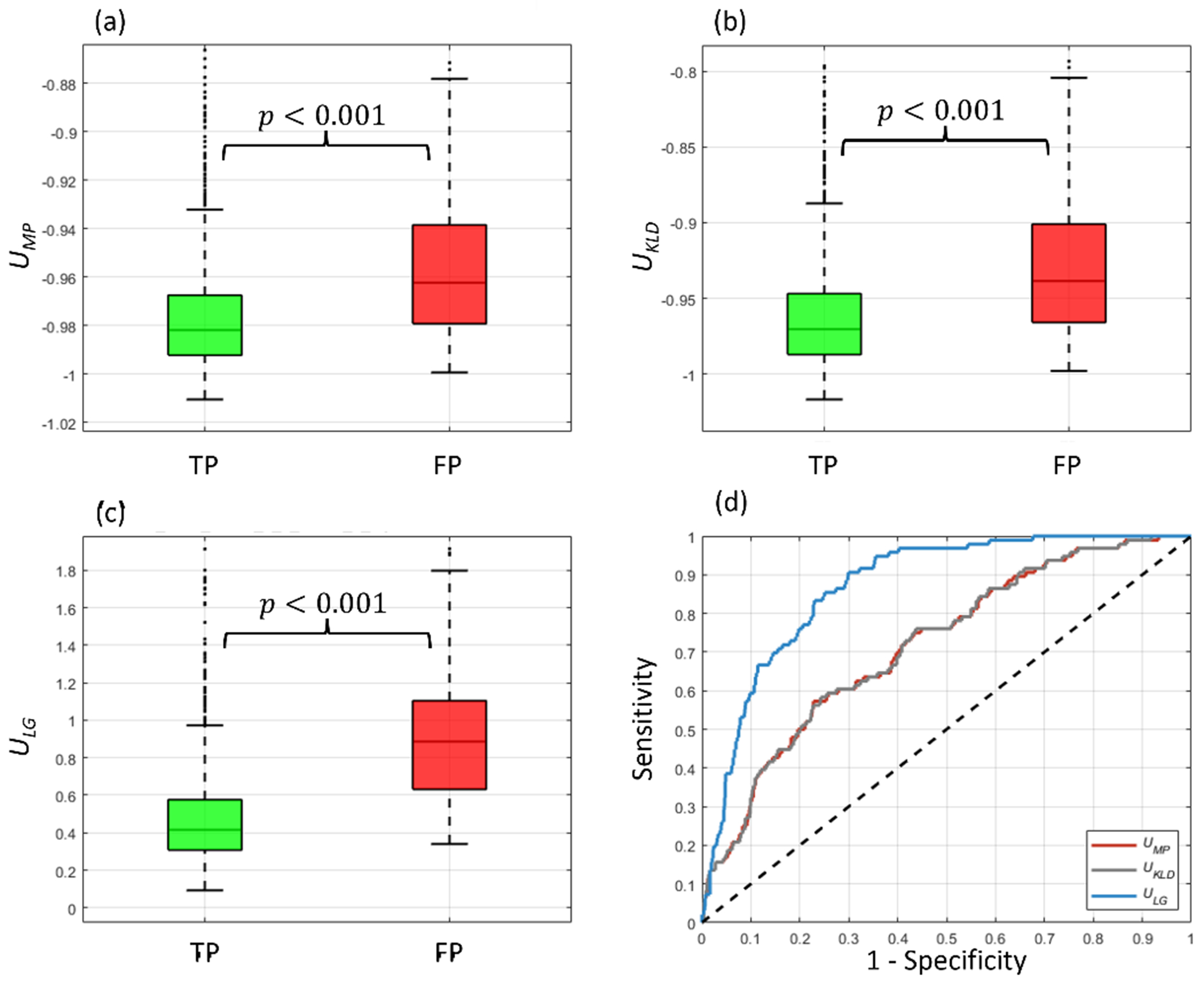
TP and FP distributions of the three tested predicted region uncertainty measures: mean probability (a), KL divergence (b), and local gradients UQ (c). (d) The ROC for filtering-out FP predicted regions using each uncertainty measure.

Figure 8 shows the distribution of TP and FP predicted regions for the *U*_MP_, *U*_KLD_, and *U*_LG_ uncertainty measures and the ROC curves of each measure for filtering FPs from TPs, where the *U*_LG_ uses gradient information from the optimal block configuration (decoder Block_4-0 and Block_4-1). All three uncertainty measures yielded statistical significance between TP and FP groups. The *U*_LG_ measure, however, achieved superior FP filtering performance over *U*_MP_, and *U*_KLD_, increasing AUC by 21% and decreasing FPR95 by 26%.

### 3.4. Correspondence with physician-rated disease likelihood

**Figure 9.**
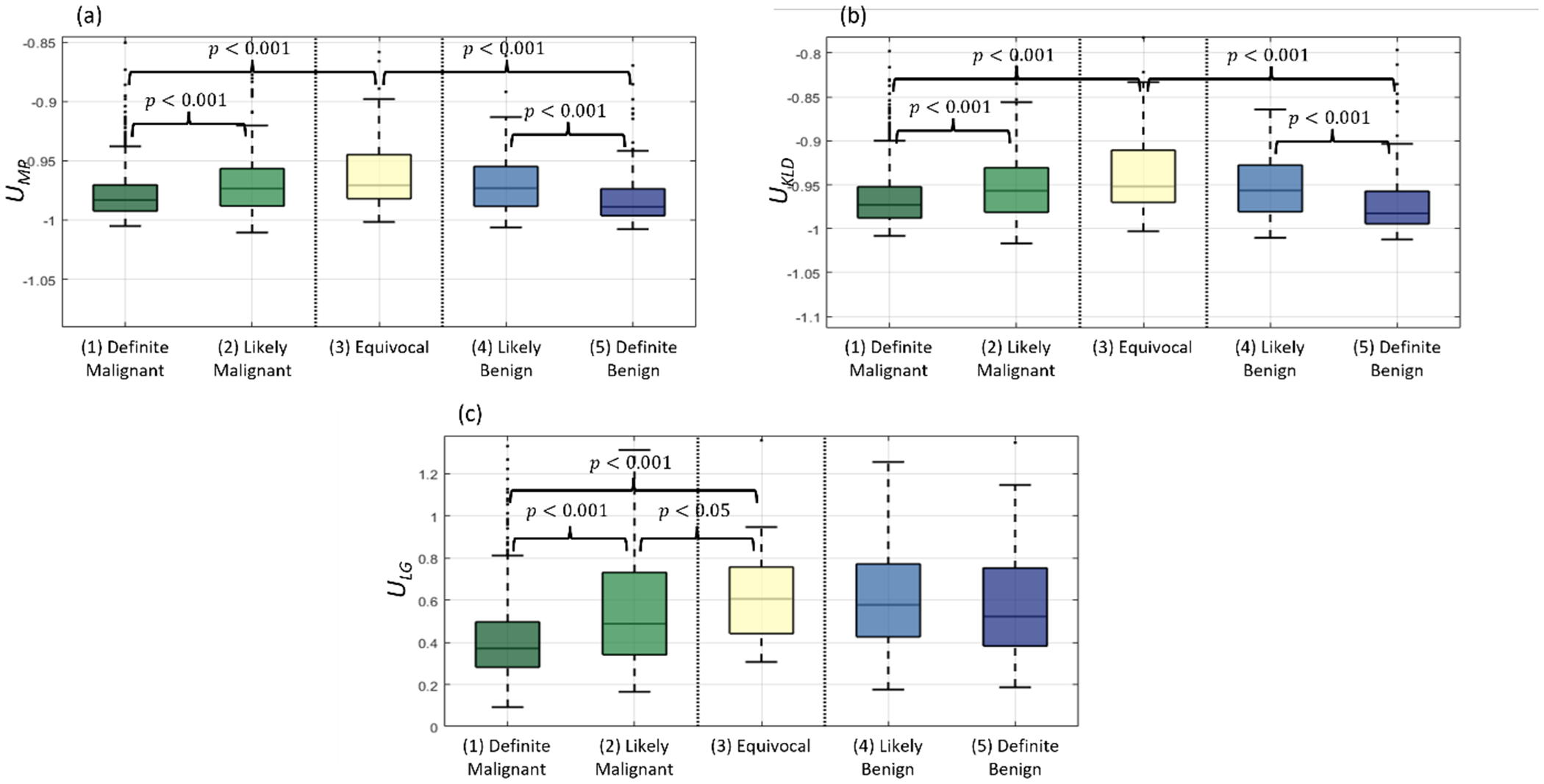
Correspondence between uncertainty measures and physician-rated disease likelihood. Predicted regions were matched to the 5-class ground-truth data of physician likelihood classifications. Results are shown for the (a) mean probability (U_MP_), (b) KL divergence (U_KLD_), and (c) local gradients (U_LG_) UQ measures.

The correspondence between the tested uncertainty measures and physician-rated disease likelihood is shown in figure 9. Statistical separation between classes in the malignant (i.e. classes 1, 2, and 3) and benign groups (i.e. classes 3, 4 and 5) for each tested uncertainty measure is reported in table 3. According to the AUC scores, the median percent differences, and *p*-values between paired groups, the *U*_LG_ measure achieved the best separation between disease classes and correspondence to physician-rated disease likelihood for the malignant classes. This is not the case, however, for the benign classes where both the *U*_MP_ and *U*_KLD_ achieved better AUC scores and *p*-values. The median percent differences across benign groups were still better for the *U*_LG_ measure.

## 4. Discussion

In this work, we introduced a novel, gradient-based method to quantify the uncertainty of regional outputs for deep learning-based medical image assessments. We demonstrated the utility of this UQ method using the application of metastatic disease delineation. We used four uncertainty validation assessments: (1) response to artificially degraded image quality, (2) comparison between matched high- and low-quality clinical images, (3) false positive (FP) filtering, and (4) correspondence with physician-rated malignant disease likelihood. In all experiments, comparisons were drawn between the novel UQ measure (*U*_LG_), and measures based on the mean probability of a predicted region (*U*_MP_) and the KL divergence of a predicted region without the use of gradients (*U*_KLD_). Overall, our results indicate that leveraging the gradient space of a trained model is useful for UQ. This agrees with the work of (Huang et al 2021), which leveraged gradients for out-of-distribution detection (OOD) for one-dimensional natural image classification models. In their work, however, a small gradient score was associated with high uncertainty (or high OOD likelihood), opposite to our formulation. This difference may be due to our localization of regional outputs for gradient computation, which may more appropriately model sensitivities of a specific regional outputs to learnt model parameters (equation (1)).

In the image degradation experiment, all three uncertainty measures increased with increasing image degradation magnitude across degradation types. Thus, all three measures followed the expected behavior and displayed some level of reliability. When compared to the non-gradient measures, the *U*_LG_ measure exhibited a stronger response to image degradation, indicating its potentially higher sensitivity to abnormal image information. This may be advantageous in a clinical setting where a trained model may encounter a wide array of abnormal image information such as artifacts which would merit human intervention. At the same time, a highly sensitive uncertainty measure may excessively flag regions as abnormal, potentially slowing down the image analysis process. This potential advantage of the *U*_LG_ measure should be considered within the context of the image analysis task. The work of (González et al 2022) similarly demonstrated the utility of an uncertainty measure using artificial image degradations, however, this study specifically targeted OOD uncertainty (a form of epistemic uncertainty). In contrast, we designed a method to target uncertainty more generally. While the image degradation experiments are indicative of the *U*_LG_ measure’s utility in capturing OOD uncertainty, more work should be dedicated specifically to evaluating this type of uncertainty using the Local Gradients UQ method.

The *U*_LG_ measure achieved only slight statistical significance in distinguishing between matched lesions in low-quality (AC-CT) and high-quality (CE-CT) CT images (*p* < 0.05). Meanwhile, the *U*_MP_ and *U*_KLD_ measures did not achieve statistical significance for this task (*p* > 0.05). One explanation for why this separation was not stronger is that it may not be consistently true that liver tumors are more detectable on CE-CT than on AC-CT from a PET/CT scan. For instance, in figure 7 we showed two examples of matched CE-CT and AC-CT liver tumors with overlaid *U*_LG_ measures. In the second example (figure 7(b)), our hypothesis that predicted tumor delineations in the lower quality (AC-CT) dataset will yield higher uncertainties was challenged. Disease presentation factors such as differences in tumor differentiation between scan types may explain this discrepancy. Thus, comparing uncertainty values between these high- and low-quality images from a clinical setting may be limited.

The *U*_LG_ measure outperformed the non-gradient measures in the FP filtering assessment. The benefit of the *U*_LG_ measure over the reference, non-gradient measures was perhaps the most evident in this assessment compared to the other assessments. These results support the assumption that incorrectly predicted regions contain high levels of uncertainty. This indicates the *U*_LG_ measure’s utility in removing erroneously predicted regions. The work of (Nair et al 2020) similarly showed that FP regions contain higher levels of uncertainty than TP regions when using MC dropout-based uncertainty measures. A potential drawback of these measures, however, is their voxel-wise uncertainty output which is aggregated into a region-wise measure for detection assessments. In this process, a region may falsely be designated as a FP due to a small portion of its regions containing high voxel-wise uncertainty (e.g. a non-obvious boundary). The *U*_LG_ measure, which directly generates a region-wise uncertainty output, mitigates this limitation. The sensitivity studied performed within the FP filtering assessment showed that leveraging gradient information from the last two convolutional blocks of the model (decoder Blocks 4–0 and 4–1) yielded sufficiently effective performance, indicating the selection of layers from which to acquire gradient information is important. These results are consistent with (Huang et al 2021) which implemented a similar methodology for UQ within the natural imaging domain and found that gradient information closer to the model’s output yielded superior UQ. Using gradient information close to the model output lends the added advantage of faster gradient computation times. FP filtering results were comparable for both gradient-based uncertainty measures (*U*_MP, Gradients_ and *U*_LG_), indicating the inclusion of gradient information from different regional target functions lends useful UQ.

Lastly, the *U*_LG_ measure showed superior correspondence with physician-rated disease likelihood score among malignant disease classes. However, the correspondence across benign disease classes was stronger for the non-gradient based measures. This discrepancy may be due to the imbalance in the number of lesions in the malignant and benign classes (table 1), where the model saw more malignant lesions during training, making its output more stable for this disease type. Assessments involving the association between deep learning-based uncertainty and reader-based uncertainty are not common, likely due to the burdensome challenge of acquiring reader-based uncertainty. In the work of (Klanecek et al 2023), an uncertainty measure was similarly related to qualitative reader assessments, however, these assessments consisted of the ‘acceptability’ of deep learning-based delineations. Both of these approaches are subject to reader errors and biases. This is especially true in our assessment where the distinction between malignant and benign disease may be very subtle and where classes were defined by a single reader. A previously performed inter-physician reader study on a subset of 14 patients in *Dataset 2—Bone Metastases* showed only moderate agreement in lesion classification across four physicians (Perk *et al* 2018). Lesion classifications from the single physician used in this work were correlated with the consensus of the multi-physicians in this subset of the data. Thus, we can expect a moderate amount of noise within the lesion classifications from the single physician used for training and assessment which may explain the discrepancy we observed in *U*_LG_ measure performance between malignant and benign disease classes. Performing this assessment using the concordance of the multiple readers would be more stable, however, acquiring the multi-reader classifications for the whole dataset was not possible.

The main challenge of this work is the assessment and validation of uncertainty measures. It is common practice to implement validation assessments where model-uncertainty is approached in the same manner as reader-uncertainty. Thus, we assume there to be a strong correlation between model- and reader-uncertainty. While this assumption lends interpretability, it may not entirely hold. More work should be dedicated to understanding the nuances of model-uncertainty and to developing standardized assessments. While the assessments in our work also base model-uncertainty on reader-uncertainty, we included several assessments in this work to test the robustness of the developed *U*_LG_ measure. Another challenge of this work is the unbounded nature of the Local Gradients UQ method. To account for this and to enhance the interpretability across uncertainty measures, we normalized the Local Gradients UQ measures to the 95th percentile of a set of scores of predicted regions with assumed low uncertainty. This normalization should be performed separately for each trained model, where it is required to identify predicted regions with assumed low uncertainty. For example, assumed low uncertainty predictions may come from regions with multi-reader confirmation or from regions with high conspicuity. However, doing so may not be straightforward or possible in certain datasets. Additionally, given the constraint of designing a post hoc method that does not change a trained model’s output, our method only acts on the model’s true and false positive output and does not allow for the recovery of false negative predicted regions. Meanwhile, other methods such as MC dropout have the potential to recover false negative regions if these regions contain high prediction uncertainty. Thus, the Local Gradients UQ method may not be optimal for certain applications. Lastly, the Local Gradients UQ method calculates gradients via backpropagation for each predicted region. As a result, the computation time is not consistent across test images and scales with the number of predicted regions within an image. Additional memory may also be required for this method due to running backpropagation upon inference.

The Local Gradients UQ method could readily be applied to other deep learning medical image tasks, especially those with ways to localize model outputs. For instance, the Local Gradients UQ method could be useful for UQ in other structure delineation or detection tasks, especially for challenging structures such as the duodenum (Gibson et al 2018). The Local Gradients UQ method could also be applied to deep learning-based image registration (e.g. Dalca et al 2019), where the propagated structures from the learned displacement fields can be used to localize model outputs over which to quantify uncertainty. As a final example, the Local Gradients UQ method could be useful for deep learning-based MRI-to-CT image synthesis tasks (e.g. Wang et al 2021), where UQ on structures that are difficult to synthesize can be acquired given pre-existing contours for model output localization. It is most natural to apply the Local Gradients UQ method to deep learning tasks with spatially representative outputs such as in the examples described above. For computer vision tasks with 1-D outputs, such as image classification, other methods, such as the one proposed by (Huang et al 2021), might be more appropriate.

Careful protocols should be established for how any uncertainty measure is to be used in deployed clinical settings. For instance, some level of human monitoring and intervention would likely be necessarily embedded into automated region delineation-based workflows. Here, displaying an uncertainty measure with each predicted region would be helpful so that the user can more quickly review predictions that derive from abnormal image information, remove false positive regions, and review potentially misclassified regions. Different intervention levels can be established by monitoring uncertainty levels on a priori assumed low uncertainty regions, ideally on a validation dataset. Lastly, different intervention approaches should be established for each image analysis task given the clinical use of each predictive model. More work should be dedicated to further understand how uncertainty measures can be appropriately leveraged in deployed clinical settings.

## 5. Conclusion

In summary, this work introduced the Local Gradients UQ method. We found that the localized gradient information, inherent to this method, enhanced region-based UQ across four validation assessments. These results indicate that users do not have to deviate far from their model to gather uncertainty information. The model’s own gradient space can effectively be leveraged for UQ.

## Figures and Tables

**Table 1.** Distribution of physician likelihood lesion classifications for Dataset 2—bone metastases. Lesions were classified on a 5-point scale in the original ground-truth data (a). For training the base lesion delineation model, lesion classes were condensed to a 3-point scale (b).

(a) Ground-Truth Classes				
Definitely Malignant (Class 1)	Likely Malignant (Class 2)	Equivocal (Class 3)	Likely Benign (Class 4)	Definitely Benign (Class 5)
863	212	85	213	224
(b) Training Classes				
Malignant (Class 1)		Equivocal (Class 2)	Benign (Class 3)	
1075		85	427	

**Table 2.** FP filtering performance of the local gradients UQ method when targeting gradient information from different decoder convolutional blocks (bottom). Two predicted regional target functions were tested: (1, right) using the KL divergence of probability values and (2, left) mean probability value. The performance of using the target function without gradient information is also reported (top). Bold font indicates the top performing metric.

	Regional Target Function (*T*_*R*_*)*					
	Mean Probability (MP*(x)_R_*)			KL Divergence (KLD*(x)_R_*)		
	
Targeted Decoder Conv Block(s)	*P*-Value ↓	AUC ↑	FPR95 ↓	*P*-Value ↓	AUC ↑	FPR95 ↓
	Without Gradient Information					

N/a	<0.001	0.72	0.83	<0.001	0.72	0.82

	With Gradient Information					

	U_MP,Gradients_			U_LG_		

Block 4–0	<0.001	0.85	0.69	<0.001	**0.87**	0.67
Block 4–1	<0.001	**0.87**	0.65	<0.001	**0.87**	0.64
Block 4–0, Block 4–1	<0.001	0.86	0.65	<0.001	**0.87**	0.61
Block 3–0	<0.001	**0.87**	0.68	<0.001	**0.87**	0.67
Block 3–1	<0.001	0.85	0.74	<0.001	0.86	0.70
Block 3–0, Block 3–1	<0.001	**0.87**	0.67	<0.001	**0.87**	0.66
Block 2–0	<0.001	**0.87**	0.70	<0.001	**0.87**	0.68
Block 2–1	<0.001	**0.87**	0.65	<0.001	**0.87**	0.66
Block 2–0, Block 2–1	<0.001	**0.87**	0.65	<0.001	**0.87**	0.66
Block 1–0	<0.001	0.85	0.77	<0.001	0.85	0.77
Block 1–1	<0.001	0.86	0.76	<0.001	**0.87**	0.73
Block 1–0, Block 1–1	<0.001	0.86	0.76	<0.001	0.86	0.75
Block 0–0	<0.001	0.82	0.79	<0.001	0.83	0.79
Block 0–1	<0.001	0.84	0.80	<0.001	0.84	0.79
Block 0–0, Block 0–1	<0.001	0.83	0.70	<0.001	0.83	0.79

**Table 3.** Separation between physician-rated clases of disease likelihood for each pair of classes across malignant (left) and benign (right) groups. Results are shown across the three tested uncertainty measures, U_MP_, U_KLD_ , and U_LG_. Percent differences are shown as absolute values of median percent differences. Bold font indicates the top performing metric.

	Malignant Classes			Benign Classes		
	
	1–2	1–3	2–3	3–4	3–5	4–5
	*U_MP_ (Mean Probability)*					

AUC↑	0.62	0.67	0.56	0.55	**0.72**	**0.68**
Percent difference ↑	0.009	0.01	0.003	0.002	0.02	0.02

*P*-value ↓	<**0.001**	<**0.001**	0.27	0.33	<**0.001**	<**0.001**

	*U_KLD_ (KL Divergence)*					

AUC ↑	0.62	0.68	0.56	**0.56**	**0.72**	**0.68**
Percent difference ↑	0.02	0.02	0.004	0.004	0.03	0.02

*P*-value ↓	**<0.001**	**<0.001**	0.26	**0.32**	**<0.001**	**<0.001**

	*U_LG_ (Local gradients UQ)*					

AUC ↑	**0.67**	**0.79**	**0.62**	0.54	0.59	0.56
Percent difference ↑	**0.27**	**0.50**	**0.22**	**0.05**	**0.15**	**0.10**

*P*-value ↓	<**0.001**	<**0.001**	<**0.05**	0.53	0.11	0.16

## Data Availability

The data cannot be made publicly available upon publication due to legal restrictions preventing unrestricted public distribution. The data that support the findings of this study are available upon reasonable request from the authors.
